# Supplementary material for: Are carbon emissions trading and green financial instruments synergistic? -Comprehensive quantitative research based on content analysis
Source: PLoS One. 2024 Mar 7;19(3):e0298601. doi: 10.1371/journal.pone.0298601 (PMC10919601; doi:10.1371/journal.pone.0298601)
Supplement: S1 Data — (ZIP) [file pone.0298601.s001.zip › Data/地级市Z_F工作报告.zip!/╝¬┴╓/░╫│╟╩╨/└┤╫╘╣1⁄2╓┌║┼í╛┬φ┐╦╩2╛▌═°í┐_2006.docx]

各位代表：

2006年至2010年是我市经济社会发展第十一个五年规划时期。根据《中共白城市委关于制定国民经济和社会发展第十一个五年规划的建议》，市政府制订了《白城市国民经济和社会发展第十一个五年规划纲要》（草案），已经提交大会。现在，我代表白城市人民政府，向大会作工作报告，请同“十一五”规划纲要（草案）一并予以审议，并请市政协各位委员提出意见。

　　一、“十五”时期及2005年经济社会发展回顾

　　过去的五年，白城市人民政府在市委的正确领导下，在市人大的依法监督和市政协的民主监督下，团结带领全市广大干部群众抢抓机遇，开拓进取，经济社会发展成效显著。2005年预计地区生产总值完成158亿元，比“九五”期末增长95.1%，年均增长14.3%，完成“十五”计划的117%。固定资产投资78亿元，比“九五”期末增长292%，年均增长31.4%，完成“十五”计划的158.4%。全口径财政收入实际完成13.93亿元，比“九五”期末增长169.4%，年均增长21.9%，完成“十五”计划的156.7%。农民人均纯收入2827元，比“九五”期末增长133.3%，年均增长18.5%，完成“十五”计划的94.2%。城镇居民人均可支配收入8400元，比“九五”期末增长102.2%，年均增长15.1%，完成“十五”计划的132%。

　　过去的五年是国民经济快速增长，综合实力显著增强的五年；是各项社会事业长足发展，社会全面进步的五年；是人民生活水平稳步提高，城乡面貌发生巨大变化的五年。“十五”时期前四年的工作，已向人民代表大会分别作了报告，下面重点报告2005年市政府的主要工作。

　　2005年，是在振兴东北老工业基地取得良好开局基础上加快发展的一年，全市上下强化发展措施，加快发展步伐，地区生产总值、全口径财政收入增幅、农民人均纯收入、城镇居民人均可支配收入以及各项主要经济指标均是“十五”时期的最高年份，固定资产投资增幅已升至全省中游水平。

　　——工业园区实现了“一年大变样”的预期目标。完成了一期工程4平方公里“七通一平”和二期工程4.2平方公里环路及相关配套设施建设。入园落地项目39个，计划投资50亿元，投资超亿元的项目8个，尼沃数控刀具车床、李官奇天利大豆纤维等27个项目开工建设。各县（市）的工业集中区建设也取得了较快进展。

　　——项目建设实现新突破。全力组织实施“百项工程攻坚”，加快推进项目建设。全市规划了138个重点项目，总投资271亿元。开工建设了九新集团白城庭峰公司玉米酒精、洮北华能风电、镇赉黑大豆深加工、洮南高档精纺呢绒、通榆风机塔筒、大安精制页岩油等投资千万元以上重点项目155项，其中亿元以上49项，完成投资52.6亿元。

　　——工业经济持续增长。预计规模以上工业增加值完成20.3亿元，比上年增长11.6%；实现销售收入52.4亿元，比上年增长18.4%。县域工业活力增强，非国有工业增势明显，新建企业增产潜力较大，有力地拉动了全市工业经济增长。

　　——农业经济取得可喜成果。粮食产量达到45.7亿斤，是白城历史上第二个丰收年，是战胜’98特大洪灾以来的第一个丰收年。农产品加工企业发展到771户，裕丰、丰盛米业等14户企业被评为省级农业产业化重点龙头企业。新建、续建牧业园区64个，成立了市奶业协会，提高了奶牛业的组织化程度，奶牛饲养量比上年增长47%。“瀚海桑田工程”新植桑树13.4万亩，桑田总面积达到20万亩；全市造林37.5万亩。水利建设完成综合工程量284万立方米。我市被列为全国第一个地级中小城市生态环境建设实验区。农村劳务输出35万人（次），创收10.1亿元。

　　——商贸经济发展迅速。预计社会消费品零售总额73.5亿元，比上年增长12%。外贸进出口额完成4300万美元，比上年增长78.3%，其中出口3900万美元，比上年增长93.9%。实际到位外资613万美元，比上年增长五倍。全省第一个绿豆出口集团公司—吉林省德泰绿豆产业基地集团公司在白城挂牌。

　　——改革工作取得重大进展。我市列入省改制计划的68户国有工业企业已基本完成改制。同时，完成了并轨任务。全市共解除劳动关系8.8万人，完成省计划的146%。基本完成了免征农业税综合配套改革试点任务。粮食流通体制改革取得了新进展。其它方面的改革有序推进。

　　——招商引资取得新成果。坚持企业为主与政府推动相结合，组织参加了“西洽会”、东北亚投资贸易博览会和赴“两省一市”经贸交流与招商等活动，积极拓展渠道，全方位招商引资。全市完成招商项目609个，到位资金69亿元，同比增长53.3%。

　　——旅游开发步伐加快。加强旅游基础设施建设，完善景区景点的整体功能。组织开展了中国·白城《向海自然保护区》特种邮票首发式暨湿地鹤乡文化旅游节活动。向海和莫莫格两个自然保护区通过了国家4A级景区评定，为我市旅游业向更高层次发展提供了有利条件。全市接待域外游客89万人（次），创收3.2亿元。

　　——基础设施建设成效显著。全市投融资39亿元，完成与市民生活密切相关的居住、出行、供水、供热等利民工程157项。“引嫩入白”工程渠首泵站基坑完成土方56万立方米。白城至石头井子高速公路市区绕越段工程前期完成投资1.1亿元；省道科铁线二级公路已经竣工；建设村村通等级公路1500公里。启动了白城机场选址、论证等工作。

　　——各项社会事业全面发展。着力推进科技创新，实施国家和省级科技发展项目108项，新建科技专家大院、农业科技示范园区18个。基础教育水平明显提高，高考文、理本科进线率分别居全省第4位和第2位。文化体育事业蓬勃发展，我市6个剧目分别获全省第二届艺术节特等奖和一、二等奖，在全省各类体育比赛中共获奖牌56枚。广播电视节目获国家和省级一等奖20项。公共卫生体系建设不断加强。计划生育工作全面完成了省下达的计划任务。在全国第一次经济普查中，我市被评为国家级先进单位。其它社会事业也取得了新成绩。

　　——城乡社会保持稳定。广泛开展“创业促就业”活动，多领域开辟就业渠道，新增就业岗位5.6万个，就业空间不断扩大。加强社会保障工作，养老、失业保险金发放率均达到100%。深入开展专项整治，进一步加强了安全生产工作。认真落实信访工作领导责任制和包案责任制，妥善解决群众反映的热点、难点问题。积极开展“平安白城”创建工作，加强社会治安综合治理，维护了社会稳定。

　　——精神文明和民主法制建设深入推进。大力开展群众性精神文明创建活动，城乡居民整体素质和文明程度不断提高。认真执行人大决议和决定，定期向人大常委会报告工作，积极支持政协和各民主党派、工商联参政议政。承办人大代表建议46件、政协委员提案和建议案69件，全部办理完毕。加大普法宣传教育和依法治市工作力度，认真贯彻《全面推进依法行政实施纲要》和《行政许可法》，积极开展行政许可项目清理，加强行政执法检查和监督，促进依法行政。

　　——政府自身建设进一步加强。积极推进政府机构改革，调整完善了政府有关部门的职能和职责。按照省政府的部署，向县（市）下放行政审批权258项。结合保持共产党员先进性教育，扎实开展了“四服务”活动。完善了政府工作制度，增强行政效能。大力推进“阳光政务”，努力营造良好的投资环境。充分发挥市长公开电话的作用，受理群众投诉和建议4616件，出现场处理156次，群众投诉办结率和满意率分别达到98.2%和96.3%。认真落实廉洁自律各项规定和“一岗双责”制，有效开展经济监督和领导干部经济责任审计，强化专项治理，廉政建设取得了新进展。

　　五年来所取得的成绩，是全市各级干部和广大群众克难攻坚、拼搏奋斗的结果，是社会各界、各方面大力支持的结果。在此，我代表市政府向全市工人、农民、知识分子、各阶层群众和各级干部，向各级人大、政协、民主党派、工商联、人民团体及各界人士，向驻白部队、武警官兵、公安司法干警和中省直驻白单位，向对我们工作给予关心和帮助的离退休老领导、老同志表示衷心的感谢和崇高的敬意！

　　同时，我们也清醒地看到制约全市经济社会发展的明显问题，主要是：经济总量小、工业基础薄、农业产业化水平低、财政收入少、投融资能力弱、科技人才缺等。全市GDP、全口径财政收入、固定资产投资仅占全省的4.7%、3.3%和4. 5 %；规模以上工业增加值占全省的3.7%，占全市GDP的37%，比全省平均水平低10个百分点；农畜产品加工转化率为35%，比全省平均水平低5个百分点；专业技术人才仅占全市人口的4.2%。对上述问题，我们要高度重视，采取着实措施认真加以解决。

　　二、“十一五”时期的主要目标任务及2006年的重点工作

　　按照市委三届九次全会的部署，“十一五”时期全市经济社会发展的指导思想是：坚持以邓小平理论和“三个代表”重要思想为指导，全面贯彻落实科学发展观，以加快发展为目标，以改革开放和科技进步为动力，以项目建设和投资拉动为主要增长源，促进全市经济更快更好的发展。切实转变发展观念、创新发展模式、加快发展速度、提高发展质量，充分发挥比较优势，做大做强支柱产业，加快推进工业化、城镇化和农业现代化。加强和谐社会建设，促进社会事业全面发展，推进全面建设小康社会进程，努力开创我市“十一五”加快发展的新局面。

　　“十一五”时期经济社会发展的总体目标：到2010年，地区生产总值达到400亿元，年均增长20%以上，比“十五”期末翻一番以上；固定资产累计投资850亿元，年均增长28%以上，前两年每年增长达到35%以上。资源利用效能显著增强，各项社会事业协调发展，城乡居民收入水平和生活质量不断提高，民主法制、精神文明、和谐社会建设步伐加快，全面建设小康社会取得阶段性成果。真正做到发展为了人民，发展依靠人民，发展成果由人民共享。

　　“十一五”时期的主要任务：

　　（一）加快工业园区建设，打造投资聚集平台，提升工业实力。科学规划工业园区产业布局，强化园区基础设施建设，完善综合配套服务，增强承接重大项目和聚集高新产业功能，做大做强工业园区，推进白城工业实力全面提升。

　　（二）突出投资拉动，滚动实施“百项工程攻坚”，扩充经济总量。积极谋划科技含量高、经济效益好、带动作用强的重大项目，着力实施一批亿元以上的大项目，在扩充经济总量上实现新突破。

　　（三）依托资源优势，建设六大基地，壮大产业规模。充分利用我市丰富的自然资源和现有的产业基础，重点培育壮大能源、农产品加工、汽车零部件、纺织、畜牧业、桑产业六大基地，优化产业结构，增强综合竞争力。

　　（四）抓住历史机遇，解决好“三农”问题，建设社会主义新农村。用工业化思维谋划农业发展，大力发展农畜特色产业，加快农业产业化步伐。加强农村新型经济组织建设和基础设施建设，壮大小城镇经济实力，推进扶贫攻坚，改善农村整体面貌。

　　（五）着力拓展国内外市场，积极发展服务业，营造白城发展的新优势。加快发展新兴、现代服务业，运用现代经营方式和信息技术改造提升传统服务业。整合旅游资源，打造“草原风光、湿地风貌、民族风情、风电景观”旅游品牌，构建旅游产业发展的新格局。

　　（六）深化重点领域改革，全方位扩大对外开放，增强经济发展活力。推进行政管理体制改革、农村改革、事业单位改革，发展壮大民营经济。在更大的范围、更广的领域、更高的层次上扩大开放，加大招商引资力度，积极发展对外贸易。加强经济开发区建设，谋求新的发展。

　　（七）继续加大投入，强化基础设施建设，提高加快发展的支撑能力。积极争取国家和省里的支持，加强交通、能源、水利、城建等基础设施建设，组织实施“引嫩入白”工程、风电和火电开发、霍乌白铁路、高速公路、白城机场等一批重点工程，为加快白城发展提供有力支撑。

　　（八）深入实施人才兴业战略，大力推进科技创新，转变经济增长方式。加快科技引进、消化和提高，推进产学研一体化进程。优先发展教育事业，整合教育资源，促进各级各类教育协调发展。强化培养、吸引和使用人才工作，营造人才创业的良好环境。

　　（九）坚持以人为本，努力建设和谐白城，实现和谐发展。坚持把扩大就业摆在更加突出的位置，千方百计增加就业。加强社会保障体系建设。协调发展人口、文化、卫生、体育等各项社会事业。创新社会管理体制和管理方式。强化生态建设和环境保护，构建资源节约和环境友好型社会。

　　2006年是实施“十一五”规划的起步年，也是白城加快发展的关键一年。我们要认真贯彻落实党的十六届五中全会和省委八届八次全会精神，按照市委的部署，凝心聚力，真抓实干，为完成“十一五”规划奠定坚实的基础。

　　主要预期目标：地区生产总值190亿元，同比增长20.3%；固定资产投资105亿元，同比增长35%；全口径财政收入按可比口径增长10%；城镇居民人均可支配收入同比增长9.4%；农民人均纯收入同比增长10%。

　　为了完成全年目标，主要抓好以下八项工作：

　　（一）全力推进项目建设，拉动经济快速增长。继续坚持以经济建设为中心、以项目建设为重、以工业项目为主的工作思路，全面发动，全民参与，全力推进，在项目建设上迈出更大的步伐。

　　加快推进工业园区的产业聚集。搞好产业规划布局，形成相对独立的产业园、园中园和产业集群。抓好园区基础设施建设，“七通一平”面积累计完成11平方公里，形成一个现代化工业新城的阶段性主体框架，同步完成当期的美化、绿化任务和“引洮入园”工程，完善综合功能，提升整体形象。全力组织好项目招商，着力引进关联度大的龙头项目、带动力强的加工项目、科技含量高的新兴产业项目，力争引进1—2户国际国内500强企业入园落地。推进玉米深加工、尼沃数控刀具车床、李官奇天利大豆纤维、白城大明公司轻钢结构等项目建设，抓好开工项目投产工作，形成项目规划、落地、建设、达产持续滚动推进的发展态势。同时，加快各县（市）工业集中区建设。

　　继续开展“百项工程攻坚”，加大投资拉动力度。市区实施104项重点项目建设，完成投资36.3亿元。在能源项目上，抓好洮北华能二期5万千瓦风电开发、洮北富裕风电项目续建和查干浩特5万千瓦风电项目开工；做好霍乌白铁路的前期工作；积极推进白城电场一期2×60万千瓦机组项目的前期工作，力争年底前通过核准，明年开工建设，抓好白城热电厂扩能等项目建设。积极推进石油开采，争取新打油井200眼以上。加快煤炭勘探和开发，在万宝区块新打千米以上勘探井2眼，并在洮北平台、洮南向阳等600平方公里范围内的预查区块实施勘探。同时，充分借助内蒙古霍林河、伊敏河地区的煤炭优势，推进火电项目建设和煤炭加工转化，做大能源产业。在农产品加工项目上，抓好年产3万吨秸秆燃料乙醇、15万吨特级食用酒精、燕麦种植及加工、洮北辣椒红色素提取等项目建设，变资源优势为经济优势。在汽车零部件项目上，抓好白城经济开发区汽车线束、红钻公司轿车玻璃升降器等项目建设，推进中一精锻与德国马勒公司的合资合作，积极引进战略投资者。在纺织服装项目上，抓好白城纺织公司高档织物替代进口、麻纺公司特种织物、洮北彩棉内衣等项目建设，不断扩大产业规模。在医药项目上，抓好天福元药业、白城中牧兽药GMP改造等项目建设，推进生化药、植化药、合成药的科技项目开发。在建材项目上，抓好远通公司建筑材料、福鑫公司环保塑钢、万通公司保温材料等项目建设，增强市场竞争力。

　　在抓好新上工业项目的同时，下功夫强化增产增效工作，提高工业运行质量。立足现有企业，把新增项目和产业扩能结合起来，做到培植远期税源与壮大近期税源“两手抓”，进一步激活存量，提高增产速度和经济效益。

　　（二）全面发展农业经济，推进社会主义新农村建设。按照生产发展、生活宽裕、乡风文明、村容整洁、管理民主的要求，把建设社会主义新农村的各项任务落到实处。

　　立足资源条件，优化农业结构。在稳步提高粮食综合生产能力的基础上，进一步扩大杂粮杂豆、燕麦、棉花、葵花等特色农产品种植面积；大力发展畜牧业，重点发展奶牛、肉牛、肉羊和白鹅业。抓好“瀚海桑田工程”和造林绿化，植桑17万亩，营造生态林草100万亩，努力提高经济效益和生态效益。

　　积极开展新农村建设试点，做好“三农”工作。重点加强农村道路、通讯、住房、水电等基础设施建设。逐步改善村容村貌，对柴草乱垛、厕所乱建、畜禽乱跑、粪土乱堆、垃圾乱倒等“五乱”现象进行集中治理。

　　加强水利设施建设，改善生态环境。重点抓好白城有史以来最大的水利工程—“引嫩入白”工程建设，加快机组安装、泵站场房及附属设施建设，确保年内部分机组提水见效。组织好江河堤防和水库除险加固、引蓄水、抗旱水源等工程建设，增强抵御自然灾害的能力。同时，积极推进水务管理体制改革。组织好全国中小城市生态环境实验区建设，提高生态建设水平。

　　大力开展劳务输出，多渠道增加农民收入。精心组织安排，抓好基地建设，完善服务措施，加强技能培训，推动农村劳动力转移。全年劳务输出保持35万人（次）。

　　（三）大力发展商贸经济，增强辐射带动功能。社会消费品零售总额同比增长16%；外贸进出口额同比增长18%。

　　在提升传统服务业的同时，积极发展信息、中介、咨询、社区等新兴服务业，不断探索电子商务、网上销售等现代营销方式，大力推进仓储超市、连锁经营、销售代理等新型流通业态，进一步完善市场体系。加强银企合作，增强金融服务功能。

　　在巩固原有出口市场的同时，着力发展新的贸易合作伙伴。整合出口资源，调整出口产品结构，提高技术含量和附加值。加大扶持力度，培育重点出口企业，提高自营出口能力。加快出口基地建设，以白城绿豆产业协会为依托，以吉林省德泰绿豆产业基地集团公司为载体，利用我市丰富的绿豆资源优势，推进产业化经营，扩大出口规模，提高对外贸易水平。

　　（四）继续深化改革，加快体制和机制创新。突出重点领域，推进各项改革，增强发展动力。

　　进一步巩固改革成果，集中力量推动改制企业发展壮大。围绕建立现代企业制度，规范完善法人治理结构，转换经营机制，提高企业的核心竞争力。抓好农村税费改革，落实各项政策，完善配套措施。推进事业单位改革，创新管理机制。同时，抓好其它领域的改革。

　　加快发展民营经济，推动全民创业。全面落实发展民营经济的各项政策，加强服务体系建设，支持和帮助民营企业扩大规模、提高水平、壮大实力，促进民营经济大发展。要以政策扶持，以典型引路，以着实措施推动，营造全民创业的浓厚氛围。

　　（五）广泛开展招商引资，拓展合资合作领域。全市招商引资完成118.6亿元，同比增长71.9%，其中市本级完成50亿元，同比增长129.3%。

　　主动走出去，加大宣传推介力度。积极组织参加“广交会”、“厦洽会”、第二届东北亚投资贸易博览会等经贸交流和招商活动，大力宣传白城的资源优势，宣传招商引资的优惠条件，宣传工业园区的特殊政策，宣传能让投资者动心放心的软环境，扩大白城影响。

　　创新招商机制，提高投融资水平。瞄准国际国内500强企业开展重点招商，围绕引进战略投资伙伴开展专项招商，组织精干力量到沿海发达地区开展蹲点招商，放下架子走出去开展敲门招商。招商引资工作既要采取新办法，又要肯下“笨”功夫，不断提高招商引资的成功率。

　　推进开发区建设，培育招商优势。充分发挥开发区外引内联、合资合作、示范带动作用，创新管理体制和运行机制，提升整体功能，增强招商引资和项目建设的吸引力。

　　（六）整合旅游资源，培育壮大旅游产业。全年接待国内外游客和旅游收入同比分别增长13%和10%。

　　高起点谋划，高品位开发。加强旅游资源普查，编制好《白城旅游产业开发建设规划》。加大旅游景区开发力度，进一步抓好向海、莫莫格4A级景区建设，科学布局，突出特色，协调发展。注重历史人文景区开发，丰富文化内涵。吸引域内外投资者参与景区的建设和管理，提高旅游开发水平。

　　立足资源优势，打造旅游品牌。以宽广辽阔的大草原为重点，集观光、休闲、度假为一体，打造“草原风光”品牌；以向海、莫莫格湿地为重点，集自然、生态、环保为一体，打造“湿地风貌”品牌；以地域风俗为重点，集蒙汉文化、乡村风光、北方民俗为一体，打造“民族风情”品牌；以风电场开发为重点，集壮观的风车群、交织的输变电网、开阔的大平原为一体，打造“风电景观”品牌，增强吸引力和竞争力。积极整合“游、行、住、食、购、娱”等要素，推动相关产业发展，形成“一个景区一个产业群体”的开发格局。

　　（七）以民生民利工程建设为重点，抓好关系群众利益的各项工作。从2006年至2008年，在市区全面实施民生民利工程，计划投融资119.6亿元，切实解决好市民居住、出行、吃水、供气、用热、如厕等方面问题，努力把城市建设和管理提高到一个新水平。今年计划投融资42亿元，实施6项民生民利工程（102件实事）：一是以棚户区改造为重点的人居环境建设工程，拆除棚户区平房60万平方米，规划建设230万平方米。二是以道路新（扩）建及维护、给排水、供热、燃气为重点的畅通便民工程，建设道路18条，铺装面积48万平方米，内环路以内主次干道、人行步道、小区巷道全部实现硬化。三是以环城河和园林绿化为重点的生态休闲设施建设工程，并加快改造“三场”（市民、新华、中兴广场）。四是以提高市区夜景品位和档次为重点的美化亮化工程，建设和完善霓虹灯、彩灯、射灯、轮廓灯、造型灯、路灯等设施，增设和更新街路指示牌。五是以垃圾处理、污水处理、空气净化、噪音监控为重点的环境保护工程，抓好生活、医疗垃圾处理场和污水处理厂建设，治理空气和噪音污染，提高环境质量。六是以落实“门前三包”和治理“十乱”为重点的马路市场整治工程，塑造文明、整洁、有序、和谐的城市形象。

　　加快交通设施建设。继续实施村村通等级公路工程，抓好白城至石头井子高速公路市区绕越段工程开工建设，搞好镇赉至嘎什根二级公路改造。积极推进白城机场前期工作。

　　全面落实各项就业政策，多渠道开发就业岗位，努力扩大就业。进一步完善养老、失业、医疗、工伤、生育保险制度，不断扩大社会保障覆盖面。抓好农村扶贫开发和城市扶困，尽快使城乡贫困人口脱贫。加强信访工作，积极化解社会矛盾。做好市长公开电话工作，切实为民排忧解难。建立健全突发公共事件应急机制，增强快速反应、应急处置能力。强化安全生产专项整治，坚决遏制重大事故的发生。继续开展“平安白城”创建工作，推进社会治安防控体系建设，维护社会和谐稳定。

　　（八）统筹发展各项社会事业，促进社会全面进步。广泛开展群众性精神文明创建活动，进一步提高城乡文明程度。围绕增强自主创新能力，抓好科技发展项目和科技示范园区建设，积极推进信息化建设。加强大专院校、科研机构、企业的联合协作，促进科技成果转化。巩固提高义务教育，加快发展职业教育，努力办好高等教育。大力发展文化体育事业，强化社区文化、企业文化和乡村文化建设，促进竞技体育和群众体育协调发展。推进公共卫生体系建设，提高医疗服务水平。切实抓好人口和计划生育工作，稳定低生育水平，提高出生人口素质。充分发挥广播电视宣传作用，营造良好的舆论氛围。加强国土资源管理。认真做好全国第二次农业普查。强化国防后备力量建设，开展好双拥共建活动。同时，积极做好民族、宗教、侨务、外事、地方志、防震减灾、气象、人工降雨、人防、老龄、妇女儿童、残联、红十字会等工作，推动社会事业协调发展。

　　三、切实转变政府职能，努力建设服务型政府

　　建设服务型政府是全面实施“十一五”规划、推进白城更快更好发展的重要保障。我们要坚持科学发展观，转变政府职能，创新管理方式，逐步建立行为规范、运转协调、公正透明、廉洁高效的行政管理体制。

　　更新行政观念，推进职能转变。面对新的形势和任务，政府各部门要淡化功利意识，强化责任意识，在激烈竞争中抢抓发展机遇，在奋力赶超中履行好职责。淡化管制意识，强化服务意识，真心实意服务企业、服务项目、服务投资者、服务群众，变“供给型”服务为“需求型”服务。淡化权力意识，强化法律意识，努力使政府的行政行为法制化。

　　着力改进政风，提高行政效能。坚持不懈地抓好精简会议、文件等有关规定的落实，切实改进会风和文风。深入调查研究，密切联系群众，了解基层情况，协调解决实际问题。以坚韧不拔的毅力、百折不挠的精神、脚踏实地的作风，全力以赴促发展，千方百计创一流。

　　强化民主法制建设，坚持依法行政。进一步完善向人大报告工作和向政协通报工作制度，认真办理人大代表建议和政协委员提案，自觉接受人大的依法监督和政协的民主监督。密切同各民主党派、工商联和人民团体的联系。建立健全社情民意反映制度、社会公示和听证制度，使人民群众充分享有知情权、参与权、监督权。组织实施好“五五”普法规划，加强法制宣传教育，积极开展法律服务。认真贯彻落实《全面推进依法行政实施纲要》，加大执法监督力度，提高依法行政水平。

　　规范行政行为，改善发展环境。深入贯彻落实《行政许可法》，抓好非许可项目的清理，坚决防止不依法办事、乱作为的现象发生。大力推进政务公开，强化政务大厅的职能，减少审批环节，简化审批程序。加强社会信用体系建设，打造诚信政府，创立守信企业，培养诚信公民。抓好软环境专项整治，强化监督检查，严肃查处“涉软”案件，营造良好的发展环境。

　　加强廉政建设，促进廉洁从政。认真落实党风廉政建设责任制，严格执行领导干部廉洁自律各项规定。积极开展执法监察，着力抓好以预算执行和专项资金审计为重点的经济监督和领导干部经济责任审计。加强专项治理，坚决纠正行业和部门不正之风。要增强自觉抵制各种腐朽思想侵蚀的能力，做到严于律己，勤于奉献，甘当公仆，鞠躬尽瘁地维护好、发展好人民群众的根本利益。

　　各位代表，今后五年是白城加快发展的战略机遇期，是我市发挥比较优势并大有作为的黄金发展期，事关白城美好的未来，事关全市人民的福祉，任务艰巨，责任重大。我们要在省委、省政府和市委的领导下，在市人大、市政协的监督支持下，牢记重托，奋力作为，以更大的勇气、更足的干劲、更扎实的作风，齐心开创各项工作的新局面，合力推进白城经济社会更好更快的发展！
